# Gaussian Mixture Modeling of Hemispheric Lateralization for Language in a Large Sample of Healthy Individuals Balanced for Handedness

**DOI:** 10.1371/journal.pone.0101165

**Published:** 2014-06-30

**Authors:** Bernard Mazoyer, Laure Zago, Gaël Jobard, Fabrice Crivello, Marc Joliot, Guy Perchey, Emmanuel Mellet, Laurent Petit, Nathalie Tzourio-Mazoyer

**Affiliations:** Groupe d’Imagerie Neurofonctionnelle, Centre National de la Recherche Scientifique, Commissariat à l’Energie Atomique, et Université de Bordeaux, Bordeaux, France; Bellvitge Biomedical Research Institute-IDIBELL, Spain

## Abstract

Hemispheric lateralization for language production and its relationships with manual preference and manual preference strength were studied in a sample of 297 subjects, including 153 left-handers (LH). A hemispheric functional lateralization index (HFLI) for language was derived from fMRI acquired during a covert sentence generation task as compared with a covert word list recitation. The multimodal HFLI distribution was optimally modeled using a mixture of 3 and 4 Gaussian functions in right-handers (RH) and LH, respectively. Gaussian function parameters helped to define 3 types of language hemispheric lateralization, namely “Typical” (left hemisphere dominance with clear positive HFLI values, 88% of RH, 78% of LH), “Ambilateral” (no dominant hemisphere with HFLI values close to 0, 12% of RH, 15% of LH) and “Strongly-atypical” (right-hemisphere dominance with clear negative HFLI values, 7% of LH). Concordance between dominant hemispheres for hand and for language did not exceed chance level, and most of the association between handedness and language lateralization was explained by the fact that all Strongly-atypical individuals were left-handed. Similarly, most of the relationship between language lateralization and manual preference strength was explained by the fact that Strongly-atypical individuals exhibited a strong preference for their left hand. These results indicate that concordance of hemispheric dominance for hand and for language occurs barely above the chance level, except in a group of rare individuals (less than 1% in the general population) who exhibit strong right hemisphere dominance for both language and their preferred hand. They call for a revisit of models hypothesizing common determinants for handedness and for language dominance.

## Introduction

Two prominent behavioral characteristics of humans, as compared to non-human primates, are the preponderance of right-handedness and the capacity to acquire language. Pioneer studies of language hemispheric lateralization using Wada test [1] have revealed that about 80% of right-handers (RH) have left language lateralization, a finding corroborated by recent investigations in healthy volunteers (Table 1). Such coincident high prevalence of two *a priori* unrelated phenotypes may indicate common underpinnings, but the relationship between manual preference (MP) and language lateralization must be weak and complex, because a large majority of healthy LH also has “typical” left language lateralization (about 75%, see Table 1). Actually, the most consistent finding regarding this relationship is an increased occurrence of atypical language lateralization (whether symmetrical or right-lateralized) in LH as compared to RH. This finding has been reported in both patients using Wada testing and healthy volunteers using either functional transcranial Doppler or functional magnetic resonance imaging (fMRI). It is worth noticing that both techniques gave very similar findings despite differences in instrumentation and physiological underpinnings, in language tasks, and in methods for computing an index of asymmetry (Table 1). While functional transcranial Doppler studies and some fMRI investigations [2] measured hemispheric asymmetries, others implemented a regional approach based on either one [3] or a combination of several regions of interest [4]. In addition, the term “atypical” has different meanings in these studies, since different thresholds for segregating typical from atypical subjects were used. For example, the threshold is set to 0 for most functional transcranial Doppler studies [5]–[7] while it can be 20 in fMRI investigations [2], [4]. In addition, atypical individuals can be considered as belonging to a single category [3], [5], [8], [9] or segregated as having either no clear lateralization (also referred to as ambilateral, or symmetrical) or strong right-hemisphere dominance (also named strongly-atypical) [4], [9]–[14]. To our knowledge, no study has so far investigated whether ambilaterality and right-hemisphere dominance are two different language lateralization phenotypes or extreme expressions of a continuous atypical phenotype, likely because tackling this issue requires establishing language lateralization distribution in a large sample of LH.

**Table 1:** Review of some main contributions on the relationship between handedness and language lateralization in healthy volunteers assessed by either functional Magnetic Resonance Imaging (fMRI) or functional Transcranial Doppler (fTCD).

Author [reference]	Task	Reference	Method	Lateralization index	MP (ES score)		RH	LH	RH	LH
				ROI/threshold	RH	LH	(N)	(N)	%typical	%typical
Knecht [5]	covert verbal fluency	none	fCTD	Hemisphere/0	>25	<25	195	131	94	77
Rosch [6]	covert verbal fluency	none	fCTD	Hemisphere/0	?		20		75	
Stroobant [7]	covert verbal fluency	none	fCTD	Hemisphere/0	>70		30		90	
Whitehouse [13]	covert verbal fluency	none	fCTD	Hemisphere/3 cat	>40	<40	45	30	80	67
							***290***	***161***	***90***	***75***
Badzakova [3]	covert verbal fluency	cross fixation	fMRI	Fr ROI/0	>0	<0	107	48	95	81
Berl [14]	semantic decision	reverse speech+tone	fMRI	Fr+Te ROI/9 cat	>40		118		98	
Bethmann [12]	semantic decision	letter string matching	fMRI	Fr+Te ROI/20, 3 cat	>33	<−47	26		92	
Hung-Georgiadis [11]	semantic encoding	word spacing	fMRI	Fr ROI/10	SR	SR	17	17	94	53
Pujol [10]	covert verbal fluency	none	fMRI	Fr ROI/5 cat	SR	SR	50	50	96	76
Springer [2]	semantic decision	tone decision	fMRI	Hemisphere/20	>50		50		94	
Szaflarski [4]	covert verbal fluency	finger tapping	fMRI	Combined ROI/20		<52		50		78
							***368***	***175***	***96***	***76***

ROI: region of interest used for lateralization index computation (Fr: Frontal, Te: Temporal). threshold: lateralization index value used to segregate typical from atypical language lateralization. MP: Manual Preference. ES score: Edinburgh inventory Score of right- (RH) and left-handers (LH). SR: Self-reported manual preference. N: sample size. %typical: fraction of individuals exhibiting typical left-hemisphere dominance for language. Summary statistics (means weighted by sample sizes, in bold italic) are provided for the set of reports using each method (fTCD or fMRI).

In order to better describe the relationship between MP and language lateralization, several studies have considered MP as a continuous rather than a binary variable, but such an approach has given inconsistent findings. Assessing MP strength (MPS) with the Edinburgh inventory for example, some authors reported a linear relationship between MPS and either occurrence of atypical subjects [5], [8] or language lateralization index [4]. However, others did not find such a correlation between such lateralization index and MPS [9], [15], or found one that did not survive exclusion of LH [15]. Some of these discrepancies may be due to statistical power limitations and it is noticeable that only one study in the literature examined a sample of more than 50 LH [5] but did not report on the relationship between actual values of the lateralization index and Edinburgh inventory.

The goal of the present study was thus to establish the distribution of language lateralization in a large sample of LH in order to 1- investigate whether two groups of atypical subjects could be identified, 2- compare this distribution to that of RH, and 3- examine its relationship with the MP strength distribution.

## Materials and Methods

We recruited 153 LH and 144 RH healthy volunteers, measured their manual preference strength (MPS), and evaluated their hemispheric lateralization for language with fMRI during covert production of sentences and word lists. Note that the sample of participants of this study is not representative of the general population, as it was deliberately enriched in left-handers aiming at a 50/50 ratio.

### 1. Participants

Participants were recruited within the framework of the BIL&GIN project, a multimodal imaging/psychometric/genetic database specifically designed for studying the structural and functional neural correlates of brain lateralization [16]. The Comité pour la Protection des Personnes dans la Recherche Biomédicale de Basse-Normandie approved the study protocol. All the 297 participants (152 men, 145 women) gave their informed, written consent, and received an allowance for their participation. All participants were free of brain abnormality as assessed by an inspection of their structural T1-MRI scans by a trained radiologist. Sample mean age was 25.3 years (S.D. = 6.4 years, range: [18, 57] years), and sample mean level of education was 15.6±2.3 years (range: [11], [20] years), corresponding to almost 5 years of education after the baccalaureate. Skull perimeter of each participant was measured, men having significantly larger SP than women (Men: 58.2±1.5 cm, Women: 55.6±1.4 cm, p<0.0001, Student-t test).

### 2. Participant manual lateralization

#### 2.1 Self-reported handedness

Participants were asked to report whether they considered themselves as right- or left-handed: 144 declared themselves RH (including 72 women), and 153 LH (including 73 women). Among the latter, 4 women declared themselves as converted RH. Note that all individuals who declared themselves as RH used their right hand for writing. Note also that during the fMRI tasks, LH subjects were free to choose the hand they preferred for using the response pad, and that 135 used their right hand and 18 their left-hand (including 5 women). Self-reported LH were 2.6 years younger than RH (RH: 26.5±6.2 years, LH: 23.9±8.3 years, p<0.0005, Student-t test), and had 1 year of education less than RH (RH 16.1±2.2 years, LH 15.1±2.3 years, p = 0.0002, Student-t test).

#### 2.2 Manual preference strength

**Figure 1:**
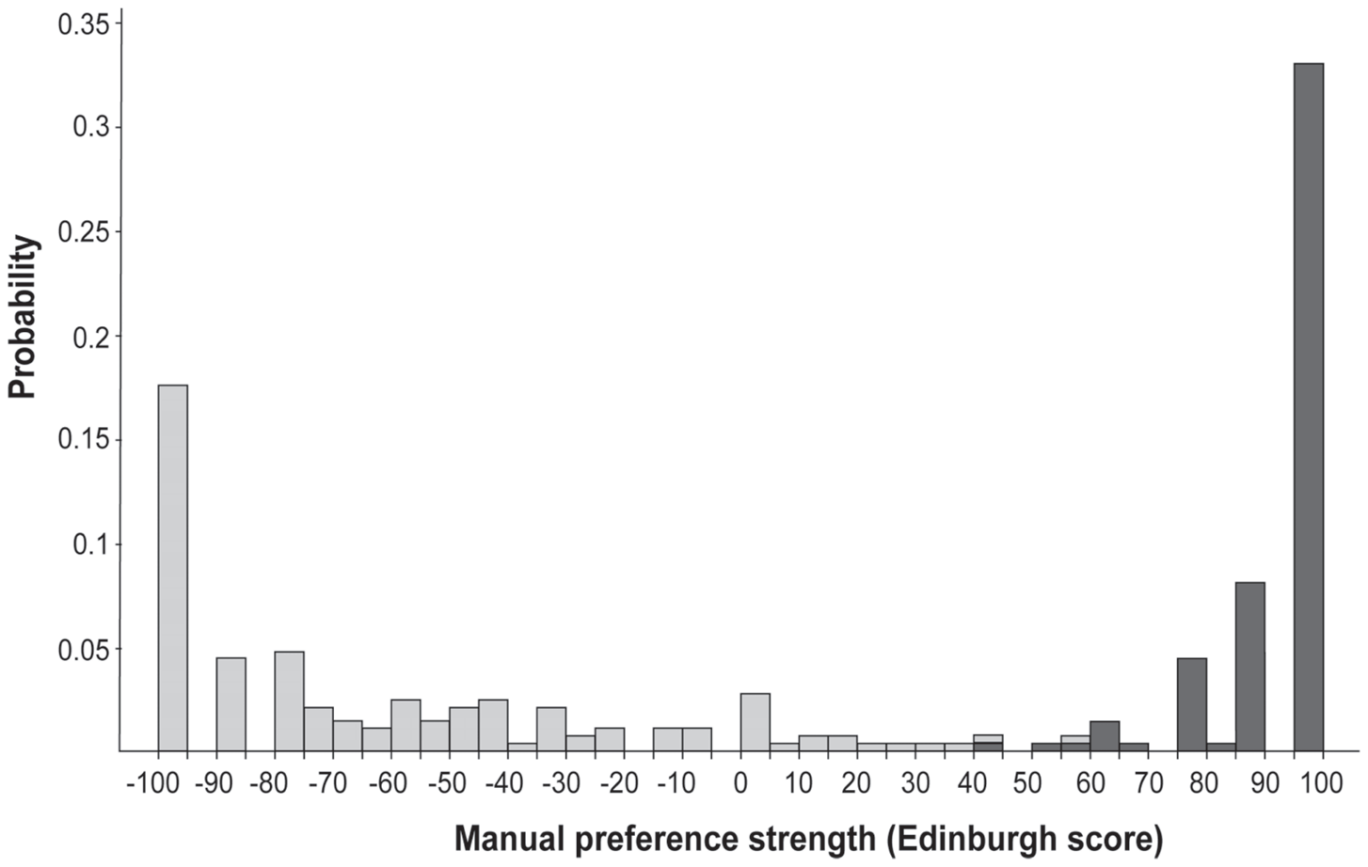
Histogram distribution of the manual preference strength variable assessed by the Edinburgh inventory score. Self-reported left- (resp. right-) handers correspond light (resp. dark) grey bars.

MPS was quantified using the score at the Edinburgh inventory [17], a series of 10 items dealing with the subject-preferred hand for manipulating objects and tools. In the present study, we only used 9 of these 10 items, dropping the “broom” item since very few young people had enough familiarity with this tool. Sample distribution of MPS is shown in Figure 1. Values ranged from −100 (strong LH) to +100 (strong RH), average MPS values being 93.1 (S.D. = 11.0) for RH, −60.0 (S.D. = 41.0) for LH subjects who used their right hand for the response pad, and −83.8 (S.D. = 22.0) for LH subjects responding with left hand, the three subgroups being significantly different from one another.

In order to compare our results with previous studies, MPS was transformed as an ordinal variable having either 3 or 7 levels, named MPS3 and MPS7, respectively. For MPS3, we used thresholds as close as possible to the 1^st^ and 2^nd^ terciles of MPS distribution. For MPS7, we used the same MPS category thresholds as previously defined by others (Knecht et al., 2000b). Values of thresholds and occurrences for each category and each variable are shown in Table 2.

**Table 2:** Boundaries and occurrences (in number of subjects (N) or fraction of the total sample size (in %) of the different manual preference strength (MPS) categories using either a 3-level (MPS3) or a 7-level scale (MPS7, as in [5]).

MPS3	[−100, −55]	[−54, 99]					100
N	99	100					98
%	33.3	33.7					33.0
**MPS7**	**−100**	**[−99, −75]**	**[−75, −25]**	**[−25, 25]**	**[25, 75]**	**[75, 99]**	**100**
N	52	31	42	24	12	38	98
%	17.5	9.1	15.5	7.7	4.4	12.8	33.0

### 3. FMRI of language production

We evaluated hemispheric dominance for language production using an index of asymmetry derived from functional MR maps contrasting covert production of sentences (SENT) with covert recitation of a list of overlearned words, namely the months of the year from January to December (LIST).

#### 3.1 Sentence and word list production tasks

**Figure 2:**
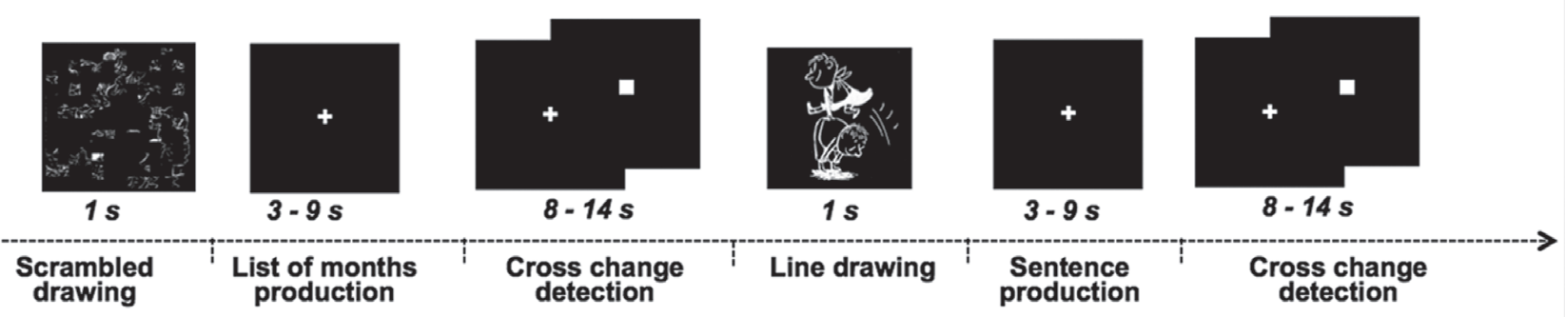
Illustration of the fMRI paradigm used for assessing language hemispheric lateralization. Subjects were presented during 1(left part) or a cartoon depicting a scene (right part). Right after presentation of a picture, the subject had to covertly generate either the list of the months of the year (right part) or a sentence describing the cartoon (left part). During this generation period, participants had to fixate a white-cross displayed at the center of the screen and to press the pad with their index finger when they had finished. Note that a reference task followed each event, consisting in sustaining visual fixation on the cross and pressing the pad when the fixation cross was switched to a square.

Subjects were presented white line drawing pictures on a black screen which were either cartoons depicting a scene involving characters, or a scrambled version of these pictures (Figure 2). Pictures covered a 14°×14° visual area and were presented for 1 sec. Right after the presentation of a picture, subjects had to covertly generate either a sentence (SENT) when they saw a cartoon or to enunciate the ordered list of the months of the year (LIST) when they saw a scrambled picture.

For SENT, subjects were instructed to generate sentences each having the same structure, starting with a subject (The little boy, The gentleman…) and a complement (with his satchel… in shorts… with glasses…), followed by a verb describing the action taking place, ending with another complement of place (in the street… in the playground… on the beach…) or of manner (with happiness… nastily…). During this generation period, participants had to fixate a white-cross displayed at the center of the screen and to press the pad with their index finger when they had finished enouncing the sentence covertly. For LIST, participants had to covertly recite the ordered list of months of the year and to press the pad when they had finished.

Note that a low-level reference task followed each event (SENT or LIST), consisting in sustaining visual fixation on the central cross and pressing the pad when the fixation cross was switched to a square (both stimulus covering a 0.8°×0.8° visual area). This second part of the trial, that lasted at least half the total trial duration, aimed at refocusing the participant attention on a non-verbal stimulus and to control for the manual motor response.

Each trial was 18 sec long, the time limit for response being 9 sec including the 1-sec picture display. A 12-sec presentation of a fixation crosshair preceded and followed the first and last trial of each run. This slow-event related experimental paradigm randomly alternated 10 trials of sentence generation with 10 trials of recitation of a list of months. Overall, the fMRI run lasted 6 min 24 sec, response time in reciting each list of words or generating each sentence being recorded using a fiber optic pad.

In order to ensure proper execution of both tasks, participants were trained outside the scanner, in the hour preceding the fMRI session. Training included both overt and covert generation of sentences and word lists, using sets of drawings that were different from those used during the fMRI session.

Right after the session, participants were asked to rate the difficulty of the task on a 5-level scale, and to recall each sentence they covertly generated during the fMRI session with the support of the pictures they saw. This makes it possible to evaluate the average number of words of covertly generated sentences for each participant. Note that the average time for sentence generation in the magnet was positively correlated with the average sentence number of words measured during debriefing (r = 0.18, p<0.0001).

#### 3.2 Anatomical and functional images acquisition

Imaging was performed on a Philips Achieva 3Tesla MRI scanner. The structural MRI protocol consisted of a localizer scan, a high resolution 3D T_1_-weighted volume acquisition (TR = 20 ms; TE = 4.6 ms; flip angle = 10°; inversion time = 800 ms; turbo field echo factor = 65; sense factor = 2; matrix size = 256×256×180; 1 mm^3^ isotropic voxel size) and a T_2_^*^-weighted multi-slice acquisition (T_2_*-FFE sequence, TR = 3,500 ms; TE = 35 ms; flip angle = 90°; sense factor = 2; 70 axial slices; 2 mm^3^ isotropic voxel size). Functional volumes were acquired with a T_2_^*^-weighted echo planar imaging acquisition (192 volumes; TR = 2 s; TE = 35 ms; flip angle = 80°; 31 axial slices; 3.75 mm^3^ isotropic voxel size) covering the same field of view than the T_2_*-FFE acquisition.

#### 3.3 Functional volume processing: individual “SENT versus LIST” contrast and t-maps

Image analysis was performed using the SPM5 software (www.fil.ion.ucl.ac.uk/spm/). The T_1_-weighted scans of the participants were normalized to our site-specific template (T_1_-80 TVS) matching the MNI space, using the SPM5 “segment” procedure with default parameters allowing for segmentation of grey matter, white matter and cerebrospinal fluid components for each participant.

In order to correct for motion during the fMRI run, each of the 192 EPI-BOLD scans was realigned to the first one using a rigid-body registration. The participant EPI-BOLD scans were then rigidly registered to his structural T_2_^*^-weighted image, which was itself registered to his T_1_-weighted scan. The combination of all registration matrices allowed each EPI-BOLD functional scan to be warped into the standard MNI space using a tri-linear interpolation, with subsequent smoothing using a 6-mm FWHM Gaussian filtering.

We then computed for each participant the BOLD signal difference map and associated t-map corresponding to the “SENT minus LIST” contrast.

#### 3.4 Probabilistic “SENT versus LIST” contrast-map in the 144 RH

In the present study, we have used a language production task (SENT) and a reference task (LIST) somewhat different from those used by previous investigators in the field (see Table 1). Accordingly, in order to document the pattern of activation elicited by SENT as compared to LIST, and specifically to show that, as expected, a left-lateralized activation in the language network was present in RH, we computed a within RH-group probability activation map using individual t-maps binarized at a 1.96 threshold.

#### 3.5 Individual Hemispheric Functional lateralization Index computation

For each individual, we computed a Hemispheric Functional Lateralization Index (HFLI) for language production (HFLI) using the LI-toolbox applied to the “SENT minus LIST” individual t-map [18]. HFLI was computed with a bootstrap algorithm using a threshold set at t = 0 (positive t-map), a lower bootstrap sample of 5 voxels and higher sample size of 1,000 voxels, and a resample ratio of *k* = 0.25. HFLI was computed within the grey and white matter anatomical template masks used for the fMRI data normalization, excluding the cerebellum. The weighted HFLI means were reported (see Table S1), values ranging between −100 and +100, with −100 being a purely right and +100 a purely left activation.

### 4. Statistical analysis

All statistical procedures were conducted using the JMP11 Pro software package, (www.jmp.com, SAS Institute Inc., 2012).

#### 4.1 Fitting HFLI sample distribution by Gaussian mixture models; definition of different types of language hemispheric lateralization

HFLI probability density function was modeled separately for LH and RH. Because of its multimodal aspect for either handedness group, a phenomenon previously noticed by others [19], HFLI probability density function was modeled using a mixture of *n* Gaussian components, namely:
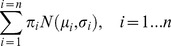
where 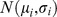 denoted the probability density function of a Gaussian distribution with mean 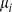 and variance 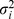, 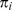 being the weight of the *i^th^* Gaussian component in the HFLI distribution (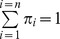). For each handedness group, the optimal model was selected as having the lowest corrected Akaike’s information criterion [20], an index that combines goodness of fit with a penalty increasing with the number of model parameters. All fits were performed using the “Continuous fit” procedure of the JMP11 Pro software, that estimates Gaussian distribution parameters 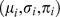 using a maximum likelihood criterion and a quasi-Newton optimization algorithm.

For both LH and RH, the optimal model, and corresponding optimal number of Gaussian functions (*n_opt_*), were then used for defining types of language hemispheric lateralization, using lower and upper HFLI thresholds best segregating the *n_opt_* Gaussian functions from each other. These thresholds were then used for assigning each participant a language lateralization type based on his individual HFLI value.

In order to compare our results, a Gaussian mixture model was also fit to the HFLI distribution observed over the entire sample of subjects.

Also, in order to compare with previous studies, we used the classical 2-category language lateralization classification obtained using a zero threshold on HFLI distribution, subjects having a positive (resp. negative) HFLI value being declared typical (resp. atypical).

#### 4.2 Comparison of behavioral variables and cognitive abilities in groups having different handedness and language lateralization types

Performances in the two tasks completed during the fMRI acquisitions were compared between groups of different handedness and language hemispheric lateralization types using an ANOVA of the response time for sentence and list generation, as well as of the mean number of words generated in sentences. Age, educational level, and sex were included as confounding factors. In order to ensure that the report made by the subject was consistent, we computed the correlation between the mean number of words per sentence and the mean time taken for their generation.

#### 4.3 Relationship between hemispheric lateralization for language and self-reported handedness

As emphasized in the Introduction section, various statistical approaches have been used for assessing the relationship between lateralization for language and manual preference, depending on whether these variables were considered as continuous, ordinal, or nominal. In the present study, we have implemented these different statistical analyses in order to be able to compare our findings with those of previous investigators and to demonstrate their robustness.

An ANOVA examined the effect of handedness on HFLI value, including sex, age, level of education and skull perimeter as confounding variables. This analysis, similar to that performed by previous investigators [4], [10], aimed at testing whether there was a significant difference in the HFLI average values between RH and LH.

Using the categorical transformation of HFLI, we also examined an association between language lateralization type and handedness (Fisher exact test). This analysis, also implemented by others [3], [13], [21], [22], aimed at testing whether there was a significant difference in the proportions of language lateralization types between RH and LH.

Finally, we implemented an original approach based on the kappa statistic [23] that is specially suited for measuring the degree of chance-corrected concordance between the hemisphere contralateral to the preferred hand (Left or non-Left) and the hemisphere dominant for language (Left or non-Left), pooling Strongly-atypicals and Ambilaterals in the same language lateralization category (non-Left dominant). This analysis aimed at testing whether there was a significant concordance in hemispheric dominance for language production and for the preferred hand in the entire sample, taking into account the concordance that one can expect by chance alone. In order to compare our results, we also measured the kappa statistic when using a 2-category language lateralization classification defined using a 0 threshold on HFLI values, as done by others [3], [9].

In order to assess the impact of Strongly-atypical subjects on the results, all analyses were repeated after excluding this subgroup from the sample.

#### 4.4 Relationship between hemispheric lateralization for language and manual preference strength

Here again, different statistical analyses were implemented with the same motivations as in the previous subsection 4.3.

- First, we studied the correlation between MPS and HFLI values as was done by others [2], [9], [11], [15]. However, because both MPS and HFLI probability density function’s markedly differed from normality, correlation between HFLI and MPS values was investigated using a Spearman rank rather than a Pearson’s correlation statistic, in each handedness group as well as in the entire sample.
- Considering the MPS3 categorical version of MPS, an ANOVA examined the effect of MPS3 on HFLI, including sex, age, level of education and skull perimeter as confounding variables. Similar analyses have been reported by others [5], [13].
- Conversely, an ANOVA examined the effect of language lateralization types (defined using the 3-category version of HFLI) on MPS values, including the same confounding variables as in b).
- Then, considering categorical versions of both HFLI and MPS, we examined an association between language lateralization and MPS3, an approach similar to that of Isaacs et al. [8].
- In addition, we also used the original approach of the kappa statistic for measuring the degree of agreement between the 3-level ordinal variables language lateralization and MPS3.
- Finally, in order to compare our results, we also conducted the same analysis than that of Knecht et al [5], searching for a linear relationship between the occurrence of atypical individuals (pooling again Ambilateral and Strongly-atypical individuals) and the 7-level ordinal version of MPS (MPS7).

As before, all analyses, except c) and e), were performed either with or without including Strongly-atypicals.

## Results

### 1. SENT versus LIST contrast activation probability map in the RH subgroup

**Figure 3:**
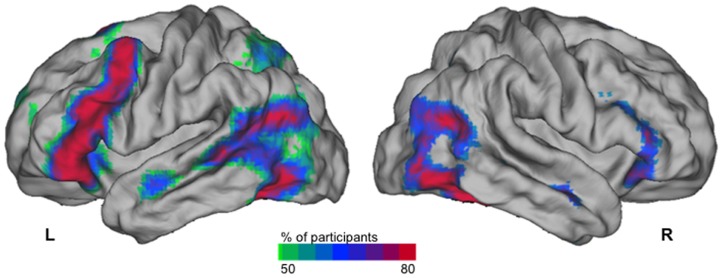
Probability map of the right-handed participants having a significant activation during sentence generation (SENT) as compared to recitation of a list of overlearned words (LIST) in the subgroup of 144 right-handers. 3D renderings of the probabilistic map of the individual SENT minus LIST contrast t-map after applying a t-threshold set at 1.96 (p<0.05, uncorrected) superimposed on the Caret anatomical template. L: left, R: right. The scale starts with 50% of overlap and the red areas correspond to a proportion larger than 80% of right-handers showing a significant activation.

The activation probability map (Figure 3) shows that more than 80% of the RH participants presented activation in the lower part of the precentral gyrus and the inferior frontal gyrus of the left hemisphere. High probability activation sites were also observed in the posterior part of the left superior temporal sulcus and its termination, in the posterior part of the left middle temporal gyrus and within the vicinity of the inferior part of the left anterior occipital sulcus. In the right hemisphere, the regions activated by at least 80% of the participants were located in the occipital lobe at the junction of the middle and inferior temporal gyri, and, to a limited extent, in the inferior frontal gyrus and anterior part of the superior temporal sulcus.

### 2. Fitting the HFLI sample distribution by Gaussian mixture models

**Figure 4:**
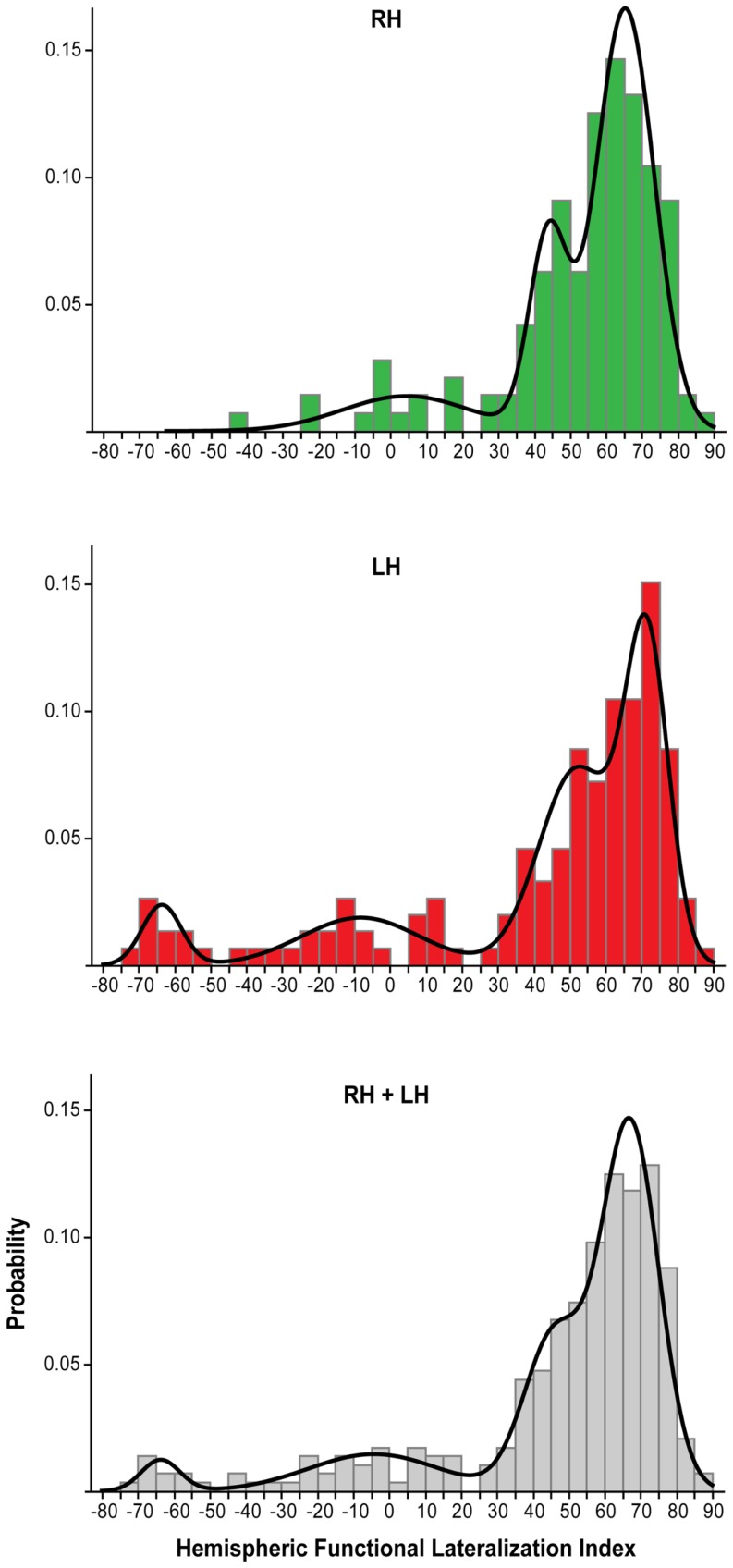
Histogram distribution of hemispheric functional lateralization index (HFLI) in right-handers (RH, top panel), left-handers (LH, middle panel) and whole sample (RH+LH, bottom panel). Solid lines are fits of these distributions by models of mixture of n Gaussian distributions (n = 3 for RH, n = 4 for LH and whole sample).

Distributions of HFLI values in LH and RH and corresponding mixture of Gaussian fits are shown in Figure 4. Both largely departed from normal, being both multimodal and skewed towards negative values because of symmetrical or rightward-asymmetrical individuals.

Optimal fits of the HFLI distributions were obtained with mixtures of 3 and 4 Gaussian functions for the RH and LH groups, respectively (Table 3). Estimates of optimal model parameters are given in Table 4 and demonstrate that HFLI distribution in RH and LH were quite similar, except for an additional Gaussian function (G4) in the model of the LH group HFLI distribution. This additional Gaussian function accounted for a small group of individuals (N = 10) having strongly negative HFLI values (average −63, range [−72, −55]). Such individuals showed strong right hemisphere lateralization for language production. Based on our sample, such a strongly atypical language lateralization seems very rare, occurring at a frequency of about 6.5% in LH only, meaning in less than 1% in the general population (assuming a 10% prevalence of LH).

**Table 3:** Gaussian mixture model fits of HFLI probability density function for the RH (top) and LH (bottom) subgroups.

n	G1	G2	G3	G4	G5	AIC_c_	Likelihood
	µ_1_,σ_1_	µ_2_,σ_2_	µ_3_,σ_3_	µ_4_,σ_4_	µ_5_,σ_5_		
	π_1_	π_2_	π_3_	π_4_	π_5_		
	**RH group**						
**1**	53.5, 22.7					1311.0	10^−24^
	100%						
**2**	60.0, 12.0	2.5, 17.1				1247.4	0.22
	89%	11%					
**3**	**65.3, 8.0**	**43.9, 5.3**	**4.4, 17.7**			**1245.9**	**1.0**
	**67%**	**20%**	**12%**				
**4**	65.5, 7.9	43.3, 6.9	5.0, 7.0	−29.3, 7.1		1253.9	3.3×10^−4^
	66%	24%	8%	2%			
**5**	65.5, 7.9	43.3, 6.9	13.3, 2.9	−2.0, 2.6	−29.3, 7.0	1252.4	1.5×10^−3^
	66%	24%	3%	4%	2%		
	**LH group**						
**1**	42.3, 40.0					1566.5	10^−60^
	100%						
**2**	62.2, 12.2	−16.2, 33.0				1442.1	10^−6^
	75%	25%					
**3**	61.3, 12.9	−7.1, 17.4	−63.6, 5.8			1434.7	2.0×10^−3^
	78%	16%	6%				
**4**	**71.5, 5.9**	**52.0, 10.6**	−**8.2, 16.4**	−**63.6, 5.4**		**1428.5**	**1.0**
	**37%**	**41%**	**15%**	**6%**			
**5**	63.8, 10.4	34.8, 3.6	10.3, 4.0	−19.2, 9.9	−63.6, 5.2	1433.2	9.1×10^−3^
	71%	8%	5%	10%	6%		

Each model is the sum of N Gaussian functions, each Gaussian function being characterized by its mean (µ_i_), standard deviation (σ_i_) and fractional contribution (π_i_) for i = 1…N. AIC_c_ is the corrected Akaike’s Information Criterion (AICc), the optimal model having the lowest AICc value. The last column indicates the relative likelihood of each model as compared to the optimal one.

**Table 4:** Comparison of HFLI optimal Gaussian mixture function model parameters for the RH and LH samples.

	G1	G2	G3	G4
	µ_1_ **[95% CΙ]**	µ_2_ **[95% CΙ]**	µ_3_ **[95% CΙ]**	µ_4_ **[95% CΙ]**
**RH**	65.3 [63.7, 66.9]	43.9 [42.0, 45.8]	4.4 [−3.8, 12.7]	–
**LH**	71.5 [69.9, 73.0]	52.0 [49.4, 54.6]	−8.2 [−14.9, −1.6]	−63.6 [−67.0, −60.3]
	σ_1_ **[95% CΙ]**	σ_2_ **[95% CΙ]**	σ_3_ **[95% CΙ]**	σ_4_ **[95% CΙ]**
**RH**	8.1 [6.7, 9.8]	5.3 [3.8, 7.4]	17.7 [12.1, 26.0]	–
**LH**	6.0 [4.6, 7.7]	10.6 [8.4, 13.4]	16.4 [11.6, 23.2]	5.4 [2.7, 10.8]
	π_1_ **[95% CΙ]**	π_2_ **[95% CΙ]**	π_3_ **[95% CΙ]**	π_4_ **[95% CΙ]**
**RH**	67.4 [52.9, 79.2]	20.3 [13.9, 28.7]	12.2 [7.5, 19.1]	–
**LH**	36.7 [27.7, 46.7]	41.3 [31.7, 51.7]	15.4 [10.1, 22.7]	6.5 [3.4, 11.9]

Each Gaussian function is characterized by its mean (µ_i_), standard deviation (σ_i_) and fractional contribution (π_i_) for i = 1…n. [95% CI] is the 95% confidence interval.

Except for these 10 subjects and the associated G4 component, HFLI distribution was characterized by 3 components for either RH or LH. The first two components (G1 and G2) gathered subjects with largely positive HFLI values, thus having a typical left lateralization for language production. These two Gaussian components, that accounted for 87.7% and 78.0% of the RH and LH probability density function, respectively, had moments that differed between the two groups. Indeed, G1 and G2 means were slightly but significantly larger in LH than in RH (71.5 versus 65.3, and 52.0 versus 43.9, respectively, p<0.05 in both cases). Meanwhile, G1 accounted for a larger fraction of the whole distribution in RH than in LH (67% versus 37%), the reverse being observed for G2 (20% for RH versus 41% in LH). As for the variance of these components, they were comparable for G1, and larger in LH than in RH for G2.

The third component (G3) appeared to have similar parameter values in both groups: a mean parameter close to 0 (4.4 in RH and −8.2 in LH), and a standard deviation around 17. This component concerned subjects with either weak or no lateralization for language production, who will be referred to as Ambilateral. Interestingly, this component represented similar fractions of the overall distribution in RH and LH (12.2% in RH and 15.4% in LH).

Fitting HFLI distribution of the entire sample of LH and RH gave results very consistent with those reported above: the optimal fit was obtained with a mixture of 4 Gaussian functions with estimated means, variance and proportions equal to (67, 46, −4, and −63), (7.9, 8.4, 19.3, and 5.3) and (57.5%, 25.7%, 13.3%, and 3.3%), respectively. Note, in particular, that, the fourth component was identical to the G4 component observed when fitting the HFLI distribution of the LH subsample (see Figure 4).

### 3. Definition and characteristics of different types of lateralization for language production

Using local minima of the optimal Gaussian mixture model function, thresholds could be easily identified for segregating the Gaussian components having the lowest HFLI means (G4 from G3 in LH, and G3 from G2 for both RH and LH, see Figure 4). On the contrary, there was a considerable overlap between the 2 Gaussian components having the largest mean values (G1 and G2 in Table 4) which led us to pool the latter two components ending with 3 subgroups having different types of language hemispheric lateralization based on their HFLI values: individuals with an HFLI positive and larger than 18 were declared Typical (130 RH, 120 LH), those with an HFLI value between −50 and 18 were declared Ambilateral (14 RH, 23 LH), and those with HFLI values below −50 were declared Strongly-atypical (10 LH). Characteristics of the three so defined language lateralization subgroups are given in Table 5 for both LH and RH. Note that, identical thresholds and classification would be observed if Gaussian component parameters had been derived from mixture modeling of the whole sample HFLI distribution for. In order to compare our results, Table 5 also reports values obtained when using the classical 2-category classification. Using the latter approach, occurrences of typical individuals reached 94.4% and 83.7% in RH and LH, respectively.

**Table 5:** Characteristics of language lateralization subjects in the RH and LH subgroups depending on their type of language lateralization using Gaussian mixture modeling (GMM, top) or a classical 2-category based on a zero threshold on HFLI values (0 THR, bottom).

GMM	RH (N = 144)			LH (N = 153)		
	Typical	Atypical	Strongly-atypical	Typical	Atypical	Strongly-atypical
**N (%)**	130 (90.3%)	14 (9.7%)	0 (0.0%)	120 (78.4%)	23 (15.0%)	10 (6.5%)
**HFLI**	59±13	−2±17	–	61±13	−9±17	−63±5
**MPS**	93±11	94±12	–	−59±42	−73±29	−87±18
**0 THR**	**RH (N = 144)**			**LH (N = 153)**		
	**Typical**	**Atypical**		**Typical**	**Atypical**	
**N (%)**	136 (94.4%)	8 (5.6%)		128 (83.7%)	25 (16.3%)	
**HFLI**	57±16	−13±15		58±18	−37±24	
**MPS**	93±11	95±14		−59±41	−82±21	

### 4. Comparison of behavioral variables and cognitive abilities in groups having different handedness and language lateralization types

Typical, Ambilateral and Strongly-atypical subjects did not significantly differ as regards their performances on tasks executed during fMRI acquisition, whether considering the number of words generated per sentence or the response time for sentence or for word list generation (see Table 6). Note that no difference was observed when classifying subjects in 2 groups, having either positive or negative HFLI during SENT. The same result was found for task difficulty that did not differ between lateralization types and did not vary with handedness. Moreover, there was no difference in performance between LH and RH, and no difference between LH using different response hands.

**Table 6:** Comparison of fMRI session performances in groups varying in manual preference or lateralization type.

Behavioral control during fMRI	RH	LH	p	Typical	Ambilateral	Strongly-atypical	p
**Self rating of task difficulty (/5)**	2.79 (0.09)	2.67 (0.09)	0.57	2.71 (0.07)	2.71 (0.18)	3.20 (0.34)	0.36
**Number of words per sentence**	12.3 (0.17)	12.5 (0.17)	0.41	12.4 (0.12)	12.0 (0.32)	13.4 (0.94)	0.17
**RT sentence production (ms)**	5597 (90)	5618 (77)	0.85	5608 (58)	5576 (167)	5676 (391)	0.94
**RT word list production (ms)**	5228 (96)	5234 (93)	0.98	5219 (70)	5363 (198)	4974 (428)	0.69

p value corresponds to the results of an ANOVA entering sex, age, SP and educational level as covariates (RT: response time, N: number). Note that there was no difference between the LH groups using different response hand.

### 5. Relationship between hemispheric lateralization for language and self-reported handedness

Average HFLI values were larger in RH (53.5±22.7, mean ± S.D.) than in LH (43.2±40.0), the difference being significant (p = 0.001, ANOVA). Note that HFLI variance was larger in LH than in RH (p<10^−4^). There was no effect of sex (p = 0.28), age (p = 0.08), educational level (p = 0.9) or skull perimeter (p = 0.12). After exclusion of the 10 Strongly-atypical subjects, average HFLI for LH was 49.8 (S.D. = 29.4) and did not significantly differ from that of RH (p = 0.11, ANOVA).

Table 7 shows the contingency table between language lateralization and handedness. A significant association was found between these two variables (Fisher’s exact test p = 0.0065) due to the fact that there was no Strongly-atypical RH. As a matter of fact, discarding the 10 Strongly-atypical subjects from the analysis, made this association no longer significant (Fisher’s exact test p = 0.11).

**Table 7:** Contingency table of language hemispheric lateralization type by self-reported handedness.

	Language lateralization type			
Handedness	Typical	Ambilateral	Strongly-atypical	All
**RH**	130 (0.44)	14 (0.047)	0 (0.00)	144 (0.49)
**LH**	120 (0.40)	23 (0.077)	10 (0.033)	153 (0.51)
**All**	250 (0.84)	37 (0.12)	10 (0.033)	297 (1.00)

Language lateralization types were derived from Gaussian mixture modeling of the probability density function of hemispheric functional lateralization index measured with fMRI. Handedness was self-reported by the subjects. RH: right-handed; LH: left-handed. Each cell contains the number of subjects and corresponding fraction of the total sample size in parentheses.

The kappa statistic was low but significantly different from 0 when considering the entire sample (κ  =  0.11, π = 0.006), but not when excluding the 10 Strongly-atypicals (κ  =  0.063, π = 0.11), meaning that agreement between hemispheric dominance for hand preference and for language was barely above the chance level. Using a 0-threshold for defining language lateralization categories gave very similar results whether including Strongly-atypicals or not (κ  =  0.105, π = 0.005, and κ  =  0.049, π = 0.13, respectively).

### 6. Relationship between hemispheric lateralization for language production and manual preference strength

**Figure 5:**
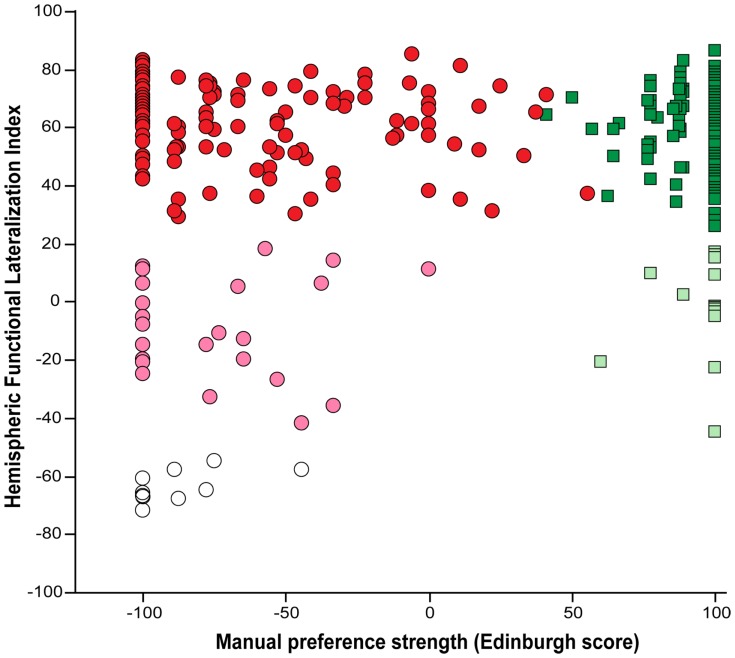
Plot of hemispheric functional lateralization for language as a function of manual preference strength. Manual preference strength was assessed using the Edinburgh inventory, ranging from 100 (exclusive use of the right hand) to −100 (exclusive use of the left hand). Subjects also self-reported whether they consider themselves as right- handed (RH, squares) or left-handed (LH, circles). HFLI, an index of hemispheric functional lateralization for language measured with fMRI during covert generation of sentences compared to covert generation of list of words, was used for classifying subjects as « Typical » (HFLI>50, bright color symbols), « Ambilateral» (−20<HFLI<50, pale color symbols), or « Strongly-atypical » (HFLI<−20, open symbols).

Figure 5 shows the plot of HFLI versus MPS values.

- Spearman rank correlation coefficient between MPS and HFLI did not significantly differ from 0, neither for RH (ρ = −0.069, p = 0.40), nor for LH (ρ = 0.059, p = 0.46), nor for the entire sample (ρ = 0.057, p = 0.32). Discarding the 10 Strongly-atypicals did not result in significant correlation (LH: ρ = −0.089, p = 0.91, RH+LH: ρ = −0.011, p = 0.84).
- A significant effect of MPS3 on HFLI values was found (p = 0.0015, ANOVA), strong LH having lower HFLI average values than either strong RH (38.3 versus 51.9, p = 0.0016) or individuals with moderate MPS (38.3 versus 53.1, p = 0.0019). This effect vanished when discarding the 10 Strongly-atypicals from the sample (p = 0.37, ANOVA).
- Conversely, a significant effect of language lateralization type on MPS values was observed (p<10^−4^, ANOVA), average MPS values of Strongly-atypicals (−87.3) being significantly different from that of either Typicals (20.0, p<10^−4^) or Ambilaterals (−9.6, p = 0.023), Typicals and Ambilaterals being also different (p = 0.01). Looking separately at RH and LH revealed that, Typical and Ambilateral RH subjects did not differ as regards their MPS average values (p = 0.60, see Table 5). Meanwhile, a significant difference was present in LH subjects between the Typical, Strongly-atypical and Ambilateral subgroups (p = 0.045, ANOVA, Table 5), due to a stronger left hand preference in Strongly-atypical LH than in Typical LH (p = 0.035, post-hoc Student’s t-test), while Ambilateral LH did not differ from either Typical LH (p = 0.12) or Strongly-atypical LH (p = 0.37).
- Consistent findings were observed when looking at the relationships between language lateralization and MPS3 categorical variables. Table 8 shows the contingency between these two variables that were found to be significantly associated (Fisher’s exact test, p = 0.001) due to the absence of individuals both strongly RH and Strongly-atypical. As a matter of fact, removing the 10 Strongly-atypical individuals from the sample turned the association to be no longer significant (Fisher’s exact test, p = 0.11). This was further confirmed when looking separately at the RH and LH groups, since non-significant association was found between language lateralization and MPS3 both for RH (Fisher’s exact test, p = 0.54) and for LH whether or not including Strongly-atypicals (Fisher’s exact test, p = 0.11 and p = 0.34, respectively).
- As for the kappa statistic, it was again small and failed to reach significance (κ  =  0.033, π = 0.055) indicating that agreement between language lateralization and MPS3 was weak and again not significantly different from the chance level.
- Finally, similar to what was found by others [5], we observed a significant linear relationship between the occurrence of atypical individuals (pooling again Strongly-atypicals and Ambilaterals) in the categories of the 7-level ordinal scale version of MPS (MPS7) and the corresponding MPS category mid-values (p = 0.030, slope = −0.089, intercept = 10.7). However, removing the 10 Strongly-atypicals from this analysis turned again this relationship to be no longer significant (p = 0.17).

**Table 8:** Contingency table of language hemispheric lateralization type by manual preference strength.

	Language lateralization type			
MPS3	Typical	Ambilateral	Strongly-atypical	All
**Strong R**	87 (0.29)	11 (0.037)	0 (0.00)	98 (0.33)
**Moderate**	90 (0.30)	9 (0.030)	1 (0.003)	100 (0.33)
**Strong L**	73 (0.25)	17 (0.057)	9 (0.030)	99 (0.33)
**All**	250 (0.84)	37 (0.12)	10 (0.033)	297 (1.00)

Language lateralization types were derived from Gaussian mixture modeling of the probability density function of hemispheric functional lateralization index measured with fMRI. Manual Preference Strength was measured with the Edinburgh inventory (MPS) and scored on a 3-level scale (MPS3). Strong R: MPS = +100, Moderate: −55<MPS<+100, Strong L: MPS<−55. Each cell contains the number of subjects and corresponding fraction of the total sample size in parentheses.

### 7. Summary of the results

In a large sample of healthy individuals, balanced for handedness, Gaussian mixture modeling of the distribution of hemispheric functional asymmetries during sentence production identified 3 types of lateralization, namely Typical (left-lateralized), Ambilateral (no lateralization) and Strongly-atypical (right-lateralized), the last category being rare (less than 1% prevalence) and including only LH. Excluding these rare subjects, we measured a concordance between dominant hemispheres for language and for the preferred hand that was not above what could be expected to occur by chance only, this being true both for RH and for LH. In LH, a significant association was observed between the strength of lateralization for language and the strength of manual preference, but this relationship was largely explained by the existence of the small group of Strongly-atypical individuals who had both strong left hand preference and strong right hemisphere dominance for language.

## Discussion

### 1. Methodological issues

Language production has long been known as the most lateralized language task as compared to speech listening that elicits smaller leftward asymmetries [7], [24], [25]. Sentence production functional asymmetries have been demonstrated to be in strong accordance with language lateralization as measured with Wada procedure [26], language tasks involving sentence leading to even more robust leftward asymmetries in healthy controls [27]. We chose to contrast the sentence production task with a high-level reference task, namely the automatic recitation of a list of overlearned words, thereby following another recommendation made by others [24]. The use of an active control task involving overlearned and automatic recitation of words in mother-tongue, and globally balanced with the sentence production task in terms of the number of words to be enounced, helped to subtract out components common to both tasks, thereby enhancing the detection of areas involved in lexico-semantic and syntactic processing.

In the present work we designed a language production paradigm that allowed for an investigation of inter-individual variability of hemispheric asymmetries of sentence processing areas. We chose to rely on a very familiar and overlearned list of words because it constitutes a high-level reference task in mother tongue that was balanced with the sentence task in terms of amount of verbal stimuli to be processed.

This paradigm allowed for obtaining robust asymmetrical contrast maps at the individual level, and its reliability is evidenced by the proportion of RH having a HFLI >0 (94.4%) strongly concordant with existing literature, and independent of the method used or of the production paradigm applied. As a matter of fact, we observed a proportion of RH with positive HFLI identical to that observed by others who used fCTD during a word fluency task (Table 1, [19]). In addition, the sentence minus list probabilistic contrast map obtained in the 144 RH illustrates the involvement of the inferior frontal gyrus and posterior STS area by this sentence production task, as well as their strong leftward asymmetry. This confirms the pertinence of the present paradigm for the determination of individual hemispheric asymmetries of language areas.

Definition of language lateralization categories in our study differs from previous works that were based on arbitrary thresholds (see Table 1). Using a large sample balanced for handedness and unsupervised Gaussian mixture modeling, we identified three non-overlapping lateralization types tailored to the multimodal nature of the HFLI distribution. This resulted in the inclusion of 19 individuals (9 RH and 10 LH) having a small but positive asymmetry index in the Ambilateral group, who would have otherwise been considered as typical. Moreover, use of Gaussian mixture modeling was also important in segregating a subgroup of individuals (Strongly-atypicals) having much lower mean HFLI than Ambilaterals.

Because typical subjects represent 90% of the population, it is important to assess whether or not they constitute a homogeneous group with respect to hemispheric dominance. Gaussian mixture model suggests the existence two distinct subgroups of typical individuals, having strong and moderate left language lateralization, respectively, this holding both for RH and for LH. However, because of the overlap between the two Gaussian distributions associated to these two putative groups (G1 and G2), it was not possible to reliably assign Typical subjects to either group. As proposed by others, additional variables, including regional patterns of functional asymmetry, may be necessary for identifying these subgroups and the factors that explain their differences [28].

Finally, it is worth mentioning that in the present studies we used two measures of handedness, namely self-report and hand preference inventory, for investigating the relationship between handedness and hemispheric dominance for language. Other measures could have been used, such as relative hand skill or performance at a reaching task. However, a recent report indicates that none of these different measures emerged as clearly superior to the others as regards their correlation with cerebral dominance for language [9].

### 2. Occurrence of the different language lateralization types in RH and LH

Using Gaussian mixture modeling-based classification, we found 90% of RH exhibiting typical language lateralization. This proportion increased to 94% with the usual binary classification based on a zero-threshold on HFLI. Such proportions are in agreement (see Table 1) with previous imaging investigations of language production dominance in RH healthy volunteers using either fMRI [2], [10] or functional transcranial Doppler [19]. With such a binary classification, atypical language lateralization occurred in 6.5% of our RH sample, identical to the 6% reported by Springer using fMRI and word generation [2], and by Mateer and Dodrill in epileptic patients using Wada testing [22].

Regarding LH, the 78% proportion of LH with typical language lateralization using the 3-group classification rose to 84% with the binary approach, identical to figures reported by Szaflarski et al. with fMRI during word production [4]. With that same binary classification, the proportion of atypical LH (16%) in our study was identical to that of Springer et al [2].

Overall, atypical language lateralization was found more frequent in LH than in RH, in agreement with pioneer neuropsychological studies conducted by Hécaen [29]. However, the difference in atypical language lateralization frequency was not significant because non-typical language lateralization frequency in RH was far from negligible, ranging in our study from 6% to 10% depending on the threshold used for segregating typical from non-typical language lateralization. Note that this result cannot be attributed to inter-individual variability in task difficulty, known to trigger increased right hemisphere attentional resources, since there was no difference in performance or in subjective task difficulty between RH and LH.

### 3. Right-hemisphere dominance for language is rare and present in LH only

Among non left-dominant language lateralization individuals, Gaussian mixture modeling segregated a subgroup of individuals with right hemisphere language dominance, confirming the existence of this rare but normal variant of language organization [5], [10], [21]. Based on the 6.5% proportion of Strongly-atypical LH observed in this study, one can estimate a 0.6% prevalence for right-hemisphere language lateralization in the general population (assuming a 10% LH prevalence). This finding is in accordance with the 8% of LH having an HFLI value below −20 in Szaflarski’s study (see Figure 2 of [4]) as well as with the 10% of LH having an HFLI value below −25 in Pujol’s study (see Table of [10]). It also fits well with a recent review of 1,208 Wada testing of epileptic patients, in which right hemisphere language dominance has been observed in 7% of LH patients free from left hemisphere damage [30].

In our sample, right-hemisphere dominance was observed only in LH, in agreement with previous studies that reported no case of rightward dominance in healthy RH subjects during verb generation [2], [10], [19]. In patients, there have been reports of aphasia after a right-hemisphere lesion in LH [29], as well as of a large prevalence of left-handedness (8 among 9) in epileptic patients with right language dominance as assessed by the Wada test [22]. Therefore, Strongly-atypical individuals appear to form a particular group characterized by joint strong right hemisphere dominance both for language and for hand, as evidenced by their −87 average Edinburgh score. Such an association between strength of left-handedness and right hemisphere language lateralization is in agreement with previous studies using either Wada test [8] or fMRI [4]. It raises the issue of a genetic origin of this rare combination of phenotypes. So far, the search for genetic variants at the origin of handedness has been unfruitful (see [31] for review). Focusing on this subgroup of Strongly-atypical subjects, using new generation sequencing techniques, might offer the opportunity of uncovering some genetic variants involved in the co-variance between handedness and language hemispheric lateralization.

### 4. Hand lateralization and language dominance are associated by chance, except in right-hemisphere dominant individuals

An important finding of our study is that, when ignoring this group of rare Strongly-atypical individuals, we found no significant chance-corrected agreement between hemispheric dominance for hand and hemispheric dominance for language production. Given the 90% of right-handedness and 90% of left-hemisphere dominance in the general population, this result may at first sight seem counterintuitive. However, one should remember that, due to this joint high prevalence, a high level of agreement between these two traits is expected due to chance only, namely in about 81% of the subjects. Other studies have already pinpointed such a lack of agreement [9], which, together with the results of the present studies appears to refute the dogma of the existence of a correlation between hemispheric dominance for language and handedness, and should lead to revisit models, including genetic ones [32], attempting to explain this association [33].

### 5. Incidence of atypical individuals and manual preference strength

Another key result of the present study is that occurrence of atypical individuals, as assessed by the 2-category classification, was found significantly correlated with strength of handedness. In a previous study, Knecht et al. [5] described a link between handedness and the occurrence of atypical individuals (defined as having negative left-minus-right laterality index as measured with fCTD during verb generation). Specifically, these authors reported an inverse relationship between MPS ranked on the same 7-level scale, and the incidence of atypical individuals across their entire study sample of both RH and LH. Although we observed a very similar relationship in terms of slope and intercept values (see section 3.5 above) when considering the whole sample of individuals, we demonstrated that this correlation 1- was present in LH but not in RH, and 2- vanished when Strongly-atypical individuals were discarded.

In order to exclude a possible dependence of this finding on the category boundaries of the MPS7 rating scale, we conducted the same analysis using a 3-level scale with almost the same number of individuals in each category (MPS3). Again, a significant relationship was found that vanished when the 10 Strongly-atypical individuals were disregarded, calling for a different interpretation of Knecht et al findings. First, one should note that the first four levels of the MP7 scale included only LH, the fifth included a majority of LH, and the last two classes included RH only (see Figure 3 of [5]). Because individual lateralization index values were not available from Knecht et al. report, we could not directly test whether discarding right hemisphere dominant individuals from their sample would render non significant the association they reported. However, based on the results of the distribution of lateralization index by MPS they reported, it is clear that strongly-atypical individuals are present in their sample, and that they are all LH and have stronger manual preference strength. Therefore, the claimed linear relationship between manual preference strength and occurrence of atypical individuals may just reflect two subgroup effects hitherto described, namely 1- larger MPS and HFLI in Strongly-atypicals as compared to Ambilateral LH, and 2-larger HFLI and MPS values in RH as compared to atypical LH. As a matter of fact, in the present study, there was no difference in MP strength or manual ability between typical and Ambilateral RH, the MP strength of Ambilateral RH being even stronger than that of typical RH.

## Conclusion

This study demonstrates that, except in a small sample of strong LH with rightward asymmetry, concordance of hemispheric dominance for hand and for language production occurs by chance. The present result thus questions the existence of a link between control of the hand and of language by the same hemisphere, while indicating that a rightward representation of language, although rare, is a normal variant of language lateralization.

## Supporting Information

Table S1 **Raw data of the study sample.**
(XLSX)
